# Real-Life Experience With Subcutaneous Plasma-Derived C1-Inhibitor for Long-Term Prophylaxis in Patients With Hereditary Angioedema: A Case Series

**DOI:** 10.3389/falgy.2022.818741

**Published:** 2022-04-11

**Authors:** Andrea Zanichelli, Chiara Suffritti, Valentina Popescu Janu, Andrea Merlo, Chiara Cogliati

**Affiliations:** UOC Medicina Generale, ASST Fatebenefratelli Sacco, Ospedale Luigi Sacco-Università degli Studi di Milano, Milan, Italy

**Keywords:** plasma-derived C1-inhibitor, hereditary angioedema, subcutaneous use, attack frequency, dose/weight ratio

## Abstract

Hereditary angioedema due to C1-inhibitor deficiency (C1-INH-HAE) is characterized by swelling attacks that may be even life-threatening. To reduce the frequency of attacks, some patients need a long-term prophylaxis (LTP). In addition to the intravenous administration, plasma-derived C1-inhibitor (pdC1-INH) has been proved effective also if administered subcutaneously at the dose of 120 IU/kg/week. In this case series, we collected from clinical records data about 5 patients with poorly controlled C1-INH-HAE with the registered LTPs or with difficult venous access, referred to the angioedema center in Milano (Italy), who received it at lower doses, i.e., 42.86–65.22 IU/kg/week. All the patients experienced a reduction in the attack rate, ranging from 29.67% to 96.53% compared with a control period with a different LTP or with no LTP. For one patient, the comparison was made with a period when he received s.c. pdC1-INH 2 (with poor outcomes) instead of 3 times a week, which made the patient experience a decrease in the attack rate from 5.26 to 1.12 attacks/month. Observation periods varied between 2.6 and 47.97 months. Two patients reported adverse events, which were localized at the infusion site and mild in severity. In conclusion, subcutaneous pdC1-INH represents an alternative therapeutic choice according to the physician's judgment for selected patients with HAE poorly controlled with registered LTPs. In patients with difficult venous access, in countries where pdC1-INH is not approved for subcutaneous administration, about half the recommended dose may be beneficial, although suboptimal results may be obtained.

## Introduction

Hereditary angioedema due to C1-inhibitor deficiency (C1-INH-HAE) is characterized by recurrent attacks of swelling that may be life-threatening in cases of laryngeal involvement. Treatment strategies for HAE include on-demand therapy to rapidly resolve angioedema symptoms, short-term prophylaxis (STP), to prevent attacks when a patient is exposed to known triggers, and long-term prophylaxis (LTP), to decrease the frequency and severity of attacks (1). Treatment must be individualized to provide optimal care and normalize quality of life. Whereas on-demand therapy is required for all patients, LTP is used as needed for individual patients.

Among the products for LTP, plasma-derived C1-inhibitor concentrate (pdC1-INH) administered intravenously was shown to reduce the frequency of acute attacks by 50% compared with placebo in a study published in 2010 (2). Despite the LTP with pdC1-INH, the patients continued to experience, on average, 6.26 attacks in 12 weeks (2), which were treated with additional rescue infusions of pdC1-INH.

Subcutaneous (s.c.) administration of Berinert^®^ (CSL Behring) has been studied in Phases 2 and 3, and open label extension COMPACT trials and shown to reduce the frequency of attacks compared with placebo (3–5). Unlike intravenous administration, s.c. administration maintained the plasma C1-INH activity levels continuously above ~40% of normal (3), which is the threshold known to have a clinically meaningful effect on preventing HAE attacks (6). In Europe, s.c. Berinert^®^ is licensed as LTP at a dose of 60 international units (IU) per kg of body weight by s.c. injection two times weekly (7, 8).

In Italy, Berinert^®^ for s.c. use has not yet been registered. Patients with severe disease and frequent attacks are prescribed other LTPs, such as intravenous pdC1-INH, androgens, and tranexamic acid. When data were collected, neither lanadelumab nor berotralstat was available in Italy. For patients experiencing frequent breakthrough attacks despite LTP or having poor venous access, an unmet need for prophylaxis is present (3).

## Method

Patients with poorly controlled C1-INH-HAE with the registered LTPs or with difficult venous access, referred to the angioedema center in Milano (Italy), were treated with plasma-derived C1-inhibitor (Berinert^®^) registered for on-demand treatment at the dose of 20 IU/kg, administered subcutaneously as LTP. The dose of s.c. pdC1-INH injection was lower than the recommended dose for subcutaneous use (which is not approved in Italy). We collected relevant data and report the results.

Demographic data and clinical history (frequency and treatment for attacks) were retrieved from the clinical records. The severity of attacks was categorized as mild if no interference with activities of daily living was experienced, moderate in case of partial interference, and severe for complete incapacity. The patients were tested for functional and antigenic C1-INH. C1-INH-HAE type 1 was diagnosed when both functional and antigenic C1-INH were ≤ 50% of normal (9).

Although the dose/weight ratio per administration is generally used in clinical studies, we chose to calculate the dose/weight ratio per week in order to allow comparisons among patients receiving a different number of administrations per week.

## Results

In this analysis, 5 patients with C1-INH-HAE type 1 were observed.

The dose/weight ratio of s.c. infusions weekly ranged from 42.86 to 65.22 IU/kg (Table 1).

**Table 1:** Patients' data.

					**Period without s.c.Berinert^®^**		**Period with s.c.Berinert^®^**			
**Pt**	**Sex**	**Age^a^**	**Weight (kg)**	**Type of HAE**	**Type of LTP/no LTP**	**Frequency of attacks/month (n. months of observation)**	**Dosage of s.c.Berinert^®^**	**Frequency of attacks/month (n. months of observation)**	**Dose/weight ratio per week (IU/kg)^b^**	**Reduction in attack rate**
1	F	23	60	1	No LTP	3 (10.78)	1,500 IU/2 tpw	2.11 (5.68)	50	29.67%
2	F	45	70	1	i.v.Cinryze^®^ 1000 IU/2 tpw	3.46 (2.6)	1,500 IU/3 tpw	0.12 (25.49)	64.29	96.53%
3	M	60	70	1	s.c.Berinert^®^ 1500 IU/2 tpw	5.26 (1.71)	1,500 IU/3 tpw	1.12 (3.58)	64.28^c^	78.71%
4	F	45	69	1	No LTP	8.80 (5.91)	1,500 IU/3 tpw	4.30 (9.76)	65.22	51.14%
5	F	49	70	1	p.o.Tranex^®^ 1 g per 3 die	2.46 (15.41)	1,500 IU/2 tpw	0.44 (47.97)	42.86	82.11%

a *age at the LTP start*.

b *120 IU per kg per week is the total recommended dose (60 IU/kg two times weekly)*.

c *42.86 in the control period*.

*LTP, long-term prophylaxis; Pt, patient; tpw, times per week*.

The frequency of attacks/month during LTP decreased in three patients (2, 4, and 5) and remained substantially stable in one patient (1) compared to the period when they were not on LTP with s.c. C1-INH. Patient 3 did not initially experience an improvement in the frequency of attacks during LTP with s.c. C1-INH administered 2 times/week (the dose weight ratio = 42.86 IU/kg per week). Therefore, the frequency of administration was increased to 3 times/week (the weekly dose/weight ratio = 64.28 IU/kg per week) and the frequency of attacks decreased from 5.26 to 1.12 attacks/month. No patient was attack free.

Additional data about the patients, laboratory findings, and attack characteristics are shown in Tables 2, 3, respectively.

**Table 2:** Laboratory findings and clinical history.

	**Pt 1**	**Pt 2**	**Pt 3**	**Pt 4**	**Pt 5**
Functional C1-INH (nv 70-130%)	9%	16%	0%	23%	20%
Antigenic C1-INH (nv 70-115%)	9%	25%	-	25%	18%
Age at diagnosis of HAE	6	32	24	8	19
Clinical history	Tonsillectomy	• Anemia • Appendectomy • Venous embolism and thrombosis of deep vessels in lower extremities • Neoplasms • Pulmonary embolism and infarction	• Diaphragmatic hernia • Hyperplasia of prostate • Diseases of esophagus, stomach, and duodenum	• Esophageal reflux • Asthma • Constipation • Other operations in the abdominal region • Diseases of esophagus, stomach, and duodenum • Acute/chronic viral hepatitis • Nephritis, nephrotic syndrome, and nephrosis • Diabetes mellitus	• Bulimia • Depressivedisorder • Anxiety

*C1-INH, C1-inhibitor; LTP, long-term prophylaxis; nv, normal values; Pt, patient*.

**Table 3:** Severity of attacks.

	**Pt 1**		**Pt 2**		**Pt 3**		**Pt 4**		**Pt 5**	
	**No LTP**	**LTP with s.c.Berinert^®^**	**LTP with i.v.Cinryze^®^**	**LTP with s.c.Berinert^®^**	**LTP with s.c.Berinert^®^ 2 tpw**	**LTP with s.c.Berinert^®^ 3 tpw**	**No LTP**	**LTP with s.c.Berinert^®^**	**LTP with p.o. Tranex^®^**	**LTP with s.c.Berinert^®^**
Severe	100%	100%	22.22%	66.67%	77.78%	25%	–	26.19%	31.58%	23.80%
Moderate	0%	0%	77.78%	33.33%	22.22%	75%	–	14.28%	28.95%	71.43%
Mild	0%	0%	0%	0%	0%	0%	–	59.52%	39.47%	4.76%

*LTP, long-term prophylaxis; Pt, patient; tpw, times per week*.

Our cohort previously received attenuated androgens, but they were discontinued because of a lack of effectiveness or adverse events.

Adverse events during s.c. administration of C1-INH were reported by 2 patients, which included erythema at the injection site and mild itching. In both cases, the adverse events were not severe and resolved spontaneously.

During LTP with s.c. C1-INH, breakthrough attacks were mostly treated at home by patients with s.c. icatibant or intravenous (i.v) C1-INH as rescue therapy. In one patient, breakthrough attacks were treated with i.v. C1-INH by health care professionals in the hospital (Table 4).

**Table 4:** Treatments used for attacks and hospital access.

	**Pt 1**		**Pt 2**		**Pt 3**		**Pt 4**		**Pt 5**	
	**No LTP**	**LTP with s.c.Berinert^®^**	**LTP with i.v.Cinryze^®^**	**LTP with s.c.Berinert^®^**	**LTP with s.c.Berinert^®^ 2 tpw**	**LTP with s.c.Berinert^®^ 3 tpw**	**No LTP**	**LTP with s.c.Berinert^®^**	**LTP with p.o. Tranex^®^**	**LTP with s.c.Berinert^®^**
Icatibant	85.71%	91.67%	0%	0%	22.22%	0%	0%	0%	100%	100%
pdC1-INH (Berinert^®^)	28.57%^a^	8.33%	0%	100%	77.78%	100%	100%	85.71%	0%	0%
pdC1-INH (Cinryze^®^)	0%	0%	100%	0%	0%	0%	0%	0%	0%	0%
Untreated	0%	0%	0%	0%	0%	0%	0%	14.28%	0%	0%
No. of attacks requiring ER/hospital access (%)	0%	0%	100%	100%	11.11%	0%	0%	9.52%	0%	0%

a *3 attacks were treated with both on-demand treatments*.

*ER, emergency room; LTP, long-term prophylaxis; pdC1-INH, plasma-derived C1-inhibitor; Pt, patient; tpw, times per week*.

## Discussion

The patients showing a reduced frequency of attacks received a mean of ~60 IU/kg per week, which is lower than the recommended weekly dose of 120 IU per kg (8). It should be noted that, in the COMPACT study (4), even the dosage of 40 IU/kg two times weekly, i.e., 80 IU/kg per week, significantly reduced the frequency of attacks compared to placebo.

The patients included in this analysis had severe disease and required LTP. Androgens were previously prescribed in our patient cohort and were discontinued because of a lack of effectiveness or adverse events. One patient switched from LTP with tranexamic acid, which was ineffective. Another patient switched from prophylaxis with i.v. C1-INH (Cinryze^®^), discontinued because of breakthrough attacks and poor venous access.

Therefore, s.c. C1-INH was prescribed to this patient cohort. The dose that proved effective in reducing the number of attacks was ~60 IU/kg per week.

The adverse events reported in this study are similar to those reported in the clinical trials COMPACT (4) and COMPACT-OLE (5), i.e., local site reactions.

The data presented in this paper are affected by some limitations. Control periods without s.c. C1-INH were usually shorter than those with s.c. C1-INH. As the attack frequency might vary during patients' lives because of unpredictable factors [i.e., stress (10)], longer observation periods could be useful to better evaluate disease severity. A wash-out period from one LTP period to another was not considered. This is a case series reporting findings obtained in 5 patients, without a control group, as we never meant to conduct neither a clinical trial nor an observation study. The therapeutic results were suboptimal, as expected, and the number of treated patients was too small to draw definite conclusions. A multicenter study with more patients would be more informative.

Far from willing to recommend a lower dosage of s.c. C1-INH, we aimed to report our real-life experience. In conclusion, even half the recommended dose of subcutaneous C1-INH may be beneficial, although suboptimal results may be obtained. These results may be useful for physicians facing patients with difficult venous access in countries where pdC1-INH is not registered for subcutaneous administration or in the absence of availability of other new drugs for long-term prophylaxis such as lanadelumab and berotralstat.

## Data Availability Statement

The raw data supporting the conclusions of this article will be made available by the authors, without undue reservation.

## Ethics Statement

Ethical review and approval was not required for the study on human participants in accordance with the local legislation and institutional requirements. The patients/participants provided their written informed consent to participate in this study.

## Author Contributions

All authors listed have made a substantial, direct, and intellectual contribution to the work and approved it for publication.

## Funding

CSL Behring funded the publishing support and journal styling services but had no role in the conduct of the research, preparation of the article, in study design, in the collection, analysis and interpretation of data, in the writing of the report, and in the decision to submit the article for publication.

## Conflict of Interest

AZ received speaker/consultancy fees from BioCryst, CSL Behring, Pharming, and Takeda. All the other authors declare that they have no financial competing interests about the topic of this article, except for the publishing support and journal styling services, which were provided by SE*Ed* Medical Publishers and funded by CSL Behring, Italy.

## Publisher's Note

All claims expressed in this article are solely those of the authors and do not necessarily represent those of their affiliated organizations, or those of the publisher, the editors and the reviewers. Any product that may be evaluated in this article, or claim that may be made by its manufacturer, is not guaranteed or endorsed by the publisher.
